# Decrease of T-cells exhaustion markers programmed cell death-1 and T-cell immunoglobulin and mucin domain-containing protein 3 and plasma IL-10 levels after successful treatment of chronic hepatitis C

**DOI:** 10.1038/s41598-020-73137-6

**Published:** 2020-09-29

**Authors:** Sylwia Osuch, Tomasz Laskus, Hanna Berak, Karol Perlejewski, Karin J. Metzner, Marcin Paciorek, Marek Radkowski, Kamila Caraballo Cortés

**Affiliations:** 1 Department of Immunopathology of Infectious and Parasitic Diseases, Medical University of Warsaw, 3c Pawińskiego Street, 02-106 Warsaw, Poland; 2 Department of Adult Infectious Diseases, Medical University of Warsaw, Warsaw, Poland; 3 Outpatient Clinic, Warsaw Hospital for Infectious Diseases, Warsaw, Poland; 4 Division of Infectious Diseases and Hospital Epidemiology, University Hospital Zurich, University of Zurich, Zurich, Switzerland; 5 Institute of Medical Virology, University of Zurich, Zurich, Switzerland

**Keywords:** Viral hepatitis, Hepatitis C, Molecular medicine, Immunopathogenesis, Adaptive immunity, Diagnostic markers

## Abstract

During chronic hepatitis C virus (HCV) infection, both CD4^+^ and CD8^+^ T-cells become functionally exhausted, which is reflected by increased expression of programmed cell death-1 (PD-1) and T-cell immunoglobulin and mucin domain-containing protein 3 (Tim-3), and elevated anti-inflammatory interleukin 10 (IL-10) plasma levels. We studied 76 DAA-treated HCV-positive patients and 18 non-infected controls. Flow cytometry measured pretreatment frequencies of CD4^+^PD-1^+^, CD4^+^PD-1^+^Tim-3^+^ and CD8^+^PD-1^+^Tim-3^+^ T-cells and IL-10 levels measured by ELISA were significantly higher and CD4^+^PD-1^−^Tim-3^−^ and CD8^+^PD-1^−^Tim-3^−^ T-cells were significantly lower in patients than in controls. Treatment resulted in significant decrease of CD4^+^Tim-3^+^, CD8^+^Tim-3^+^, CD4^+^PD-1^+^Tim-3^+^ and CD8^+^PD-1^+^Tim-3^+^ T-cell frequencies as well as IL-10 levels and increase in CD4^+^PD-1^−^Tim-3^−^ and CD8^+^PD-1^−^Tim-3^−^ T-cells. There were no significant changes in the frequencies of CD4^+^PD-1^+^ T-cells, while CD8^+^PD-1^+^ T-cells increased. Patients with advanced liver fibrosis had higher PD-1 and lower Tim-3 expression on CD4^+^T-cells and treatment had little or no effect on the exhaustion markers. HCV-specific CD8^+^T-cells frequency has declined significantly after treatment, but their PD-1 and Tim-3 expression did not change. Successful treatment of chronic hepatitis C with DAA is associated with reversal of immune exhaustion phenotype, but this effect is absent in patients with advanced liver fibrosis.

## Introduction

Hepatitis C virus (HCV) infection is a common etiologic factor of chronic hepatitis, liver cirrhosis, and hepatocellular carcinoma (HCC). The World Health Organization’s Global Hepatitis Report estimates that about 71 million individuals are currently infected with HCV worldwide^1^. The majority of infected subjects (55–80%) develop chronic infection, whereas a minority eliminates the virus spontaneously, almost exclusively in the acute phase^2^. The ultimate outcome of HCV infection is determined by the host immune response, in particular by the strength of specific CD4^+^ and CD8^+^ T-cell activity^3,4^. Reduction in viral load and elimination of HCV is related to T-cell activation, cytotoxic elimination of infected cells, and effector cytokines production^5^. However, as the HCV infection progresses, there is a gradual decrease of the effector functions of T-cells including diminished proliferative potential and cytotoxicity, and lowered IL-2, tumor necrosis factor α (TNF-α), and interferon γ (IFN-γ) production. This progressive impairment of the host immune function is referred to as immune exhaustion^6^.

T-cell exhaustion has been described in other viral infections such as human immunodeficiency virus (HIV), hepatitis B virus (HBV) as well as in the murine model infection with lymphocytic choriomeningitis virus (LCMV) and is believed to contribute to the development of chronic infection^3,7–10^. While persistent antigen exposure is thought to be the major factor driving T-cell exhaustion, regulatory T-cell activation, anti-inflammatory cytokines (e.g. IL-10) production and increased expression of inhibitory receptors (iRs) on T-cell surface are all likely to play a contributing role^11,12^. Prolonged up-regulation of multiple iRs negatively affects T-cell function by competing with co-stimulatory molecules, interfering with signals from co-stimulatory molecules or T-cell receptors (TCRs) and by upregulation of genes involved in T-cell dysfunction^13–16^. It has been shown that overexpression of iRs during an early stage of infection facilitates the development of chronic HCV infection^17^. Among known T-cell specific iRs, the best characterized are programmed cell death-1 (PD-1) and T-cell immunoglobulin and mucin domain-containing protein 3 (Tim-3)^7,18–22^.

PD-1 is expressed on activated CD4^+^ and CD8^+^ T-cells and interaction of PD-1 with its ligands results in the suppression of T-cell sensitivity to antigenic stimulation^23,24^. Tim-3 is mainly present on Th1 (helper T-cell 1) and Tc1 (cytotoxic T-cell 1) subsets, but it is also expressed on innate immune cells such as dendritic cells (DCs), natural killer (NK) cells, and monocytes^25^. The effect of its action is downregulation of IFN-γ production and apoptosis induction^26,27^. In chronic HIV, HBV, and HCV infection, elevated PD-1 and/or Tim-3 expression characterize subpopulations of both total and virus-specific exhausted T-cells^9,17,28,29^. Functional exhaustion can be reversed by blocking of interactions of iRs with their ligands^23,27,30–32^, but exclusive blockade of the PD-1 pathway with specific monoclonal antibodies does not fully restore cell functionality. McMahan et al.^17^ showed that blocking of either the PD-1 or Tim-3 pathway enhances proliferation of HCV-specific CD8^+^ T-cells in vitro, whereas cytotoxicity against a hepatocyte cell line expressing cognate HCV epitopes was increased exclusively by Tim-3 blocking. Similarly, Urbani et al. showed that while PD-1/PD-L1 blocking enhances IL-2 and IFN-γ production by HCV-specific CD8^+^ T-cells, there is no improvement of the cytolytic function^33^. However, a combination of antibodies against PD-1 and Tim-3 does restore cell function, including cytotoxity^6^. These findings are congruent with the concept of hierarchical model of functional T-cell exhaustion in which restoration of proliferation capacity is followed by restoration of effector cytokines production and only then by the restoration of cytotoxicity.

It was demonstrated that co-expression of PD-1 and Tim-3 characterizes terminally differentiated, exhausted T-cells^34–36^. In chronic viral infections, CD4^+^ and CD8^+^ T-cells with PD-1^+^Tim-3^+^ phenotype produce immunosuppressive IL-10 that protects healthy tissues from damage by activated immune cells^27,37,38^. Plasma IL-10 levels are often increased in patients with chronic HCV, HIV, or HBV infections and this could promote transition to chronic disease^39–41^. IL-10 blockade by monoclonal antibodies was found to reduce viral load and PD-1 expression in the murine model of LCMV infection^42^.

A number of studies were devoted to the characterization of markers of immune exhaustion in HCV infection and their importance in determining the infection outcome (spontaneous elimination *vs* persistence)^31,43–45^. However, it remains largely unknown whether exhaustion markers normalize after therapy-induced viral clearance. In the present study we analyzed the effect of direct acting antivirals (DAA) treatment-induced elimination of HCV on PD-1 and Tim-3 expression on peripheral CD4^+^ and CD8^+^ T-cells, including HCV-specific CD8^+^ T-cells and on IL-10 plasma levels. We found that an effective antiviral therapy of chronic HCV infection decreases the frequencies of T-cells expressing exhaustion markers and lowers plasma concentrations of IL-10, but these effects are absent in patients with advanced liver fibrosis. HCV-specific CD8^+^ T-cells frequency significantly declined after treatment, but the PD-1 and Tim-3 expression phenotype of these cells was not affected.

## Results

### The impact of treatment on PD-1 and Tim-3 expression phenotype of peripheral CD4^+^ and CD8^+^ T-cells

#### *PD-1 and Tim-3 expression phenotype of CD4*^+^*T-cells*

**Figure 1:**
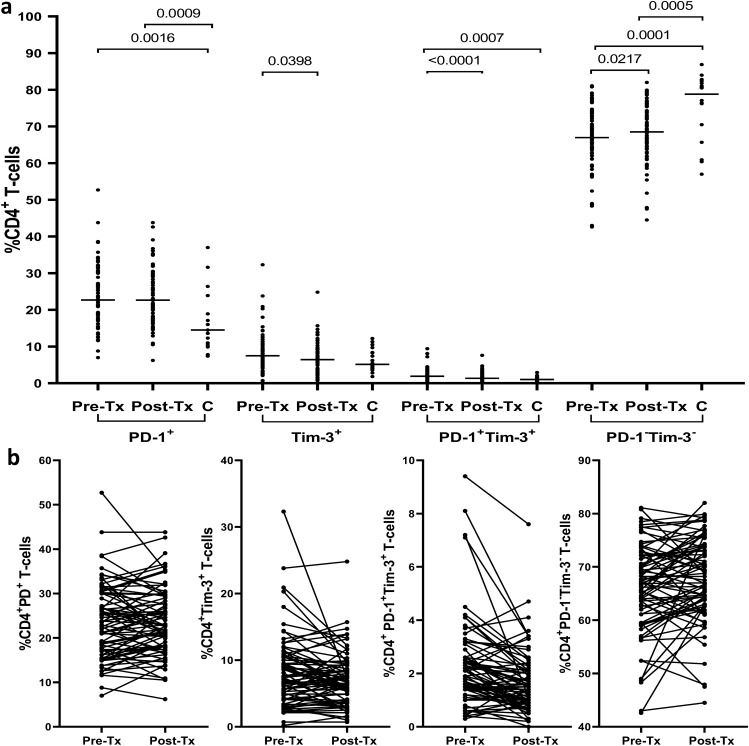
Peripheral blood CD4^+^ T-cells expression of PD-1 and Tim-3 in 76 patients before and after successful therapy of chronic hepatitis C and in 18 non-infected controls (**a**) and individual PD-1 and Tim-3 expression changes before and after treatment (**b**). Horizontal lines represent median values. Numbers above each brace express P values. *Pre-Tx* before therapy, *Post-Tx* after therapy, C- uninfected controls.

Pretreatment frequencies of CD4^+^ T-cells expressing PD-1 and PD-1 + Tim-3 were found to be significantly higher in HCV-infected patients than in controls (median 22.7% (range 7.0–52.7%) *vs* 14.5% (7.4–37.0%), P = 0.0016 and 1.9% (0.3–9.4%) *vs* 1.0% (0.3–2.9%), P = 0.0007, respectively); (Fig. 1). The pretreatment frequencies of CD4^+^ T-cells expressing Tim-3 were also higher in HCV-positive patients, although not statistically significant (7.5% (0.2–32.3%) *vs* 5.1% (1.8–12.2%). In contrast, the pretreatment frequencies of CD4^+^ expressing neither PD-1 nor Tim-3 were significantly lower in patients than in controls (66.9% (42.6–81.1%) *vs* 78.8% (57.0–86.9%), P = 0.0001).

Treatment resulted a significant decrease of CD4^+^Tim-3^+^ T-cells frequencies from 7.5% (0.2–32.3%) to 6.4% (0.7–24.8%), P = 0.0398 and CD4^+^ PD-1^+^Tim-3^+^ T-cells from 1.9% (0.3–9.4%) to 1.3% (0.0–7.6%), P < 0.0001; (Fig. 1). The frequency of CD4^+^ cells expressing PD-1 did not change (22.7% (7.0–52.7%) *vs* 22.6% (6.2–43.8%)). In contrast, CD4^+^PD-1^−^Tim-3^−^ T-cells increased significantly from 66.9% (42.6–81.1%) to 68.5% (44.5–82.0%), P = 0.0217.

**Figure 2:**
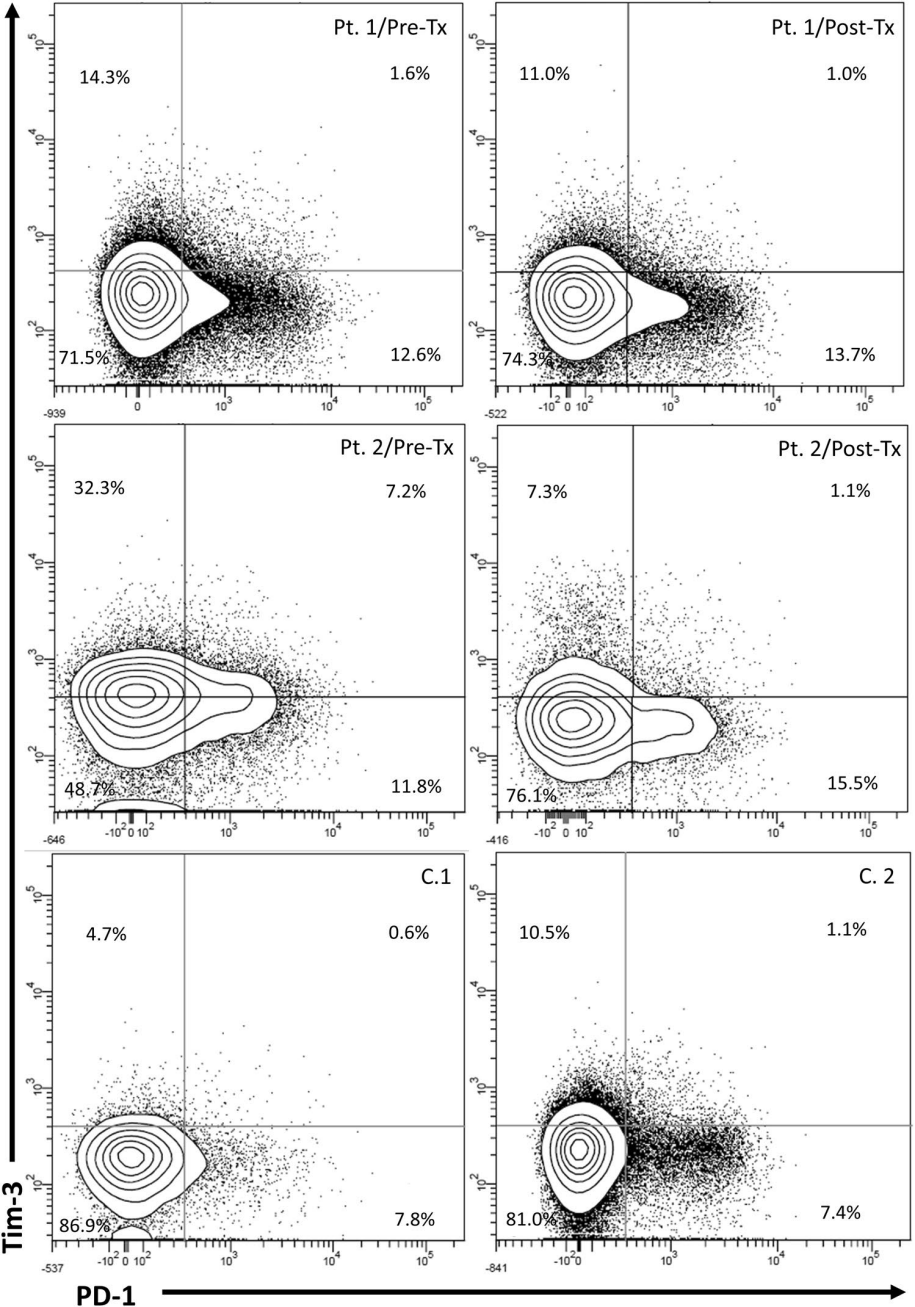
Representative cytometric analysis of peripheral blood CD4^+^ T-cells expression of PD-1 and Tim-3 before and after successful therapy for chronic HCV infection in Patient 1 (Pt. 1), Patient 2 (Pt. 2) and in two uninfected controls (C.1 and C.2). *Pre-Tx* before therapy, *Post-Tx* after therapy.

After therapy, with the exception of CD4^+^PD-1^+^ and CD4^+^PD-1^−^Tim-3^−^ T-cells (22.6% (6.2–43.8%) *vs* 14.5% (7.4–37.0%), P = 0.0009 and 68.5% (44.5–82.0%) *vs* 78.8% (57.0–86.9%), P = 0.0005, respectively), frequencies of all other analyzed subpopulations did not differ significantly from those in controls. A representative cytometric analysis of treatment-related changes in the expression of exhaustion markers on CD4^+^ T-cells in two patients and two controls is shown in Fig. 2.

#### *PD-1 and Tim-3 expression phenotype of CD8*^+^*T-cells*

**Figure 3:**
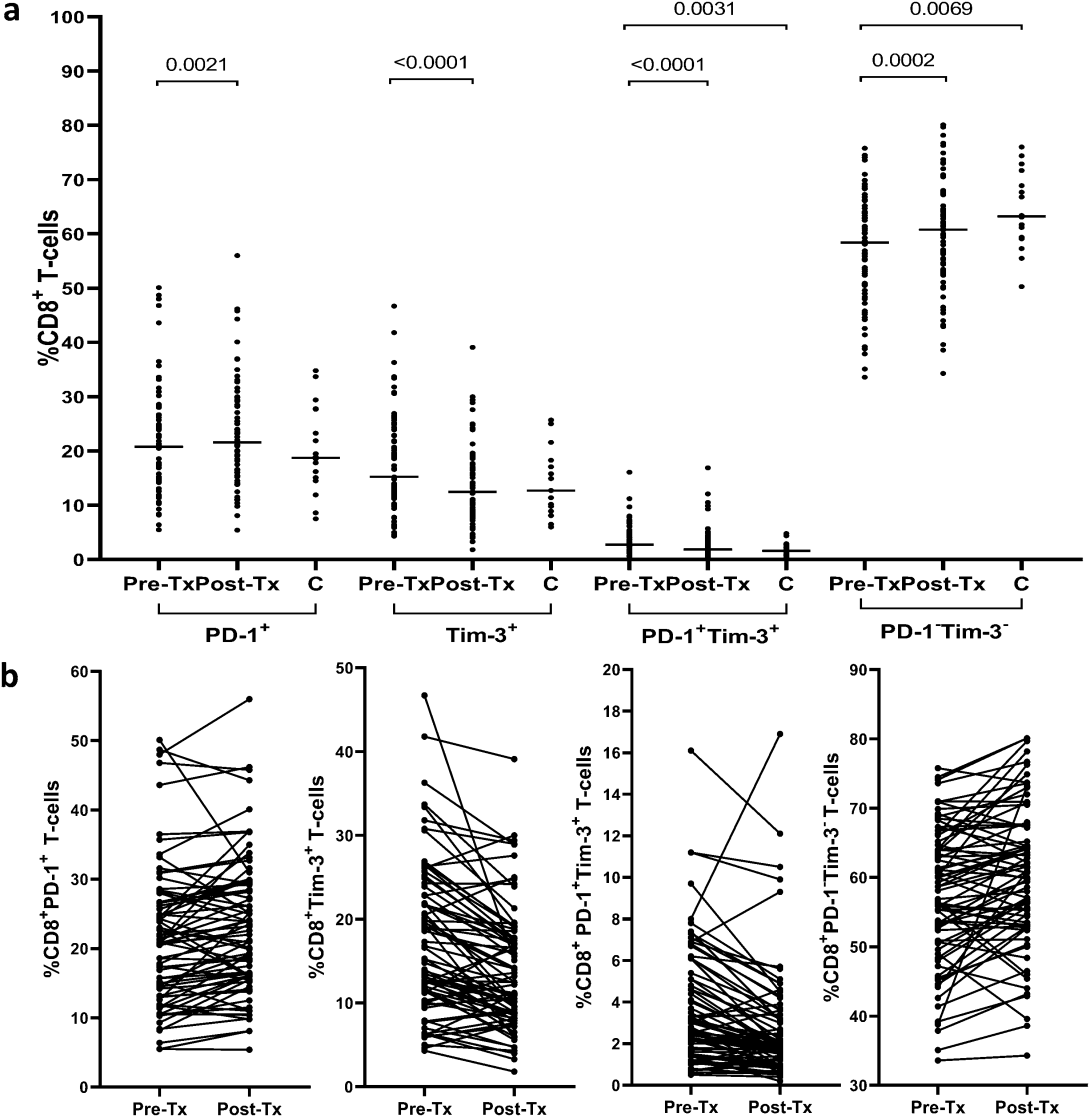
Peripheral blood CD8^+^ T-cells expression of PD-1 and Tim-3 in 76 patients before and after successful therapy for chronic HCV infection and in 18 non-infected controls (**a**) and individual PD-1 and Tim-3 expression changes before and after treatment (**b**). Horizontal lines represent median values. Numbers above each brace express P-values. *Pre-Tx* before therapy, *Post-Tx* after therapy, *C* non-infected controls.

Pretreatment frequencies of CD8^+^ T-cells co-expressing PD-1 and Tim-3 were significantly higher in patients than in controls (median 2.7% range (0.5–16.1%) *vs* 1.6% (0.3–4.8%), P = 0.0031; Fig. 3). The pretreatment frequencies of CD8^+^ T-cells expressing PD-1 or Tim-3 were also higher in patients, although not statistically significant (20.8% (5.5–50.1%) *vs* 18.7% (7.5–34.8%) and 15.2% (4.3–46.7%) *vs* 12.7% (6.0–25.7%), respectively). In contrast, the pretreatment frequencies of CD8^+^ expressing neither PD-1 nor Tim-3 were significantly lower in HCV-positive patients than in controls (58.4% (33.6–75.8%) *vs* 63.2% (50.3–76.0%), P = 0.0069).

As shown in Fig. 3, treatment resulted in a marked decrease of CD8^+^ T-cells expressing Tim-3 from 15.2% (4.3–46.7%) to 12.4% (1.8–39.1%), P < 0.0001 and CD8^+^ T-cells expressing PD-1 + Tim-3 from 2.7% (0.5–16.1%) to 1.8% (0.2–16.9%), P < 0.0001. In contrast, the frequency of CD8^+^ T-cells expressing PD-1 increased after treatment from 20.8% (5.5–50.1%) to 21.6% (5.4–56.0%), P = 0.0021 and so did the frequency of CD8^+^ PD-1^−^Tim-3^−^ T-cells (from 58.4% (33.6–75.8%) to 60.8% (34.3–80.1%), P = 0.0002).

**Figure 4:**
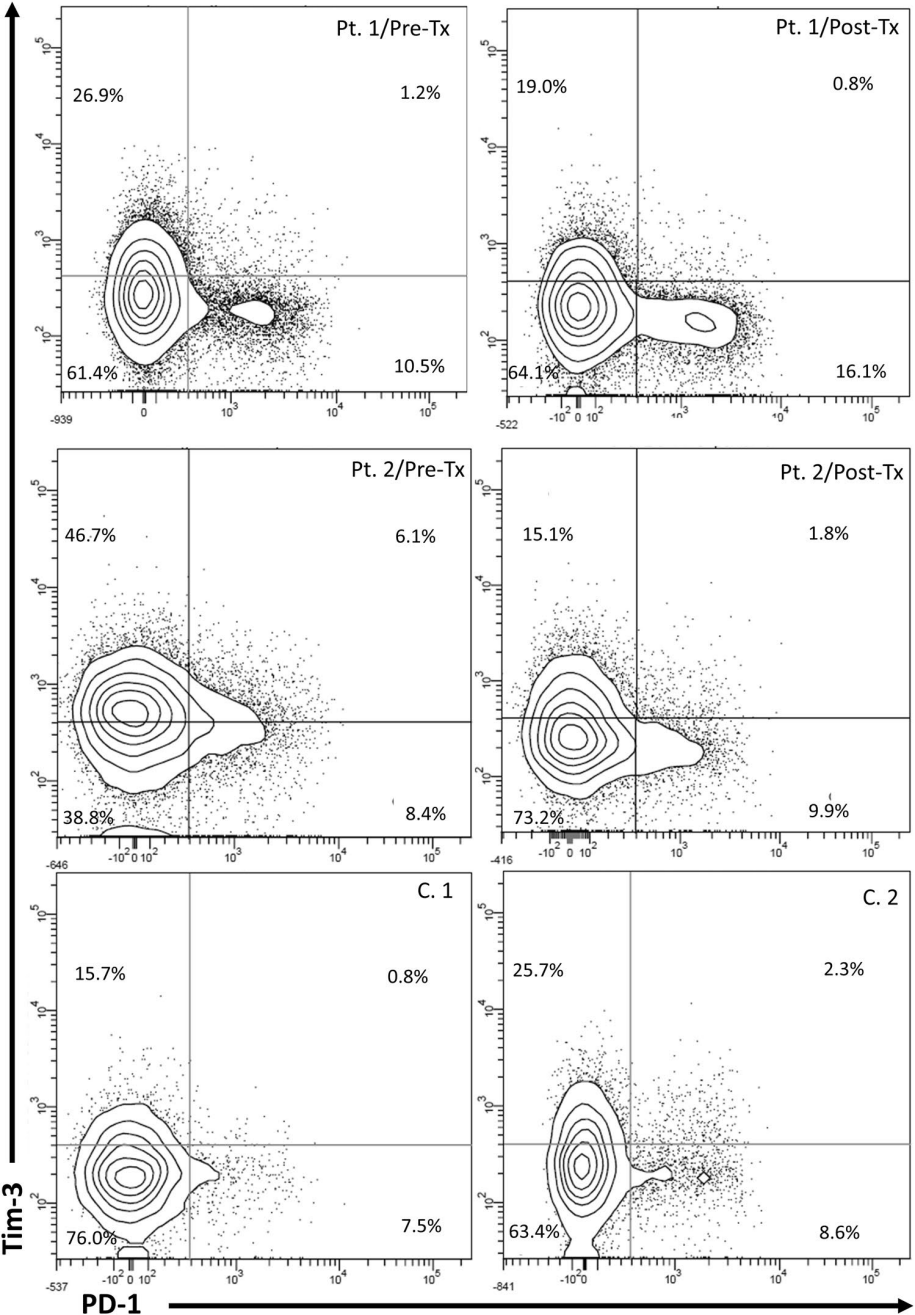
Representative cytometric analysis of peripheral blood CD8^+^ T-cells expression of PD-1 and Tim-3 before and after successful therapy for chronic HCV infection in Patient 1 (Pt. 1) and Patient 2 (Pt. 2) and in uninfected controls (C.1 and C.2). *Pre-Tx* before therapy, *Post-Tx* after therapy.

After therapy, none of the four CD8^+^ T-cells subpopulations significantly differed from those in controls (21.6% (5.4–56.0%) *vs* 18.7% (7.5–34.8%) for PD-1^+^, 12.4% (1.8–39.1%) *vs* 12.7% (6.0–25.7%) for Tim-3^+^, 1.8% (0.2–16.9%) *vs* 1.6% (0.3–4.8%) for PD-1^+^Tim-3^+^, and 60.8% (34.3–80.1%) *vs* 63.2% (50.3–76.0%) for PD-1^−^Tim-3^−^ T-cells, respectively) (Fig. 3). A representative cytometric analysis of treatment-related changes in CD8^+^ T-cells exhaustion markers in two patients and two controls is shown in Fig. 4.

### The effect of clinical and virological parameters on the pretreatment peripheral CD4^+^ and CD8^+^ T-cells PD-1 and Tim-3 expression phenotype and IL-10 plasma levels

Since pretreatment expression of exhaustion markers turned out to be highly variable within the analyzed group of patients, we tried to determine whether they were affected by clinical and/or virological parameters. Multivariate analysis included such factors as age, sex, viral load, ALT activity levels, baseline METAVIR liver fibrosis score, weight, and prior treatment (Table 1). In our analysis male sex was associated with higher IL-10 plasma levels (regression coefficient 1.54, 95% CI 0.03 to 3.04, P = 0.045) and higher percentage of CD8^+^PD-1^+^ T-cells (regression coefficient 7.69, 95% CI 2.39 to 13.00, P = 0.005). Furthermore, older age was associated with higher percentage of CD4^+^PD-1^+^ T-cells (regression coefficient 0.18, 95% CI 0.06 to 0.29, P = 0.003) and CD8^+^PD-1^+^Tim-3^+^ T-cells (regression coefficient 0.05, 95% CI 0.01 to 0.09, P = 0.025) as well as lower percentage of CD4^+^PD-1^−^Tim-3^−^ T-cells (regression coefficient − 0.18, 95% CI − 0.30 to − 0.06, P = 0.003). Importantly, when compared to F0/1, F3 liver fibrosis score was associated with higher percentage of CD4^+^PD-1^+^ T-cells (regression coefficient 5.69, 95% CI 0.13 to 11.26, P = 0.045), but the opposite was true in case of CD4^+^Tim-3^+^ T-cells (regression coefficient − 3.4, 95% CI − 6.66 to − 0.30, P = 0.032 for F2 and regression coefficient − 5.20, 95% CI − 9.09 to − 1.31, P = 0.010 for F3). Furthermore, F2 stage was associated with higher percentage of CD8^+^PD-1^−^Tim-3^−^ T-cells (regression coefficient 7.14, 95% CI 1.37 to 12.91, P = 0.016).

**Table 1:** Clinical, laboratory and virological characteristic of patients and controls.

	Patients n = 76	Controls n = 18
Age [median (range), years]	58.5 (25–88)	49.5 (23–73)
Male/female (%)	29/47 (38.2/61.8)	4/14 (22/78)
Viral load [mean ± SD, IU/mL]	1.59 × 10^6^ ± 1.31 × 10^6^	N/A
ALT activity [mean ± SD, IU/L]; (normal values ≤ 56 IU/mL)	80.1 ± 39.8	27.1 ± 19.5
Treatment scheme Ledipasvir + sofosbuvir/ombitasvir + paritaprevir + ritonavir + dasabuvir (%)	54/22 (71.1/28.9)	N/A
Treatment naïve [Y/N]	53/23	N/A
Weigh [kg]	74.4 ± 13.5	N/A
**FibroScan**^**a**^		
F0/1	38	N/A
F2	27	N/A
F3	11	N/A
F4	0	N/A

*N/A* not available or not applicable.

^a^5-point METAVIR scale was used for liver fibrosis grading where F0/F1 represents no or minimal fibrosis, F2 moderate fibrosis, F3 severe fibrosis, and F4 represents cirrhosis^46^.

### Liver fibrosis scores correlation with peripheral T-cell PD-1 and Tim-3 expression phenotype and its treatment-related change

**Figure 5:**
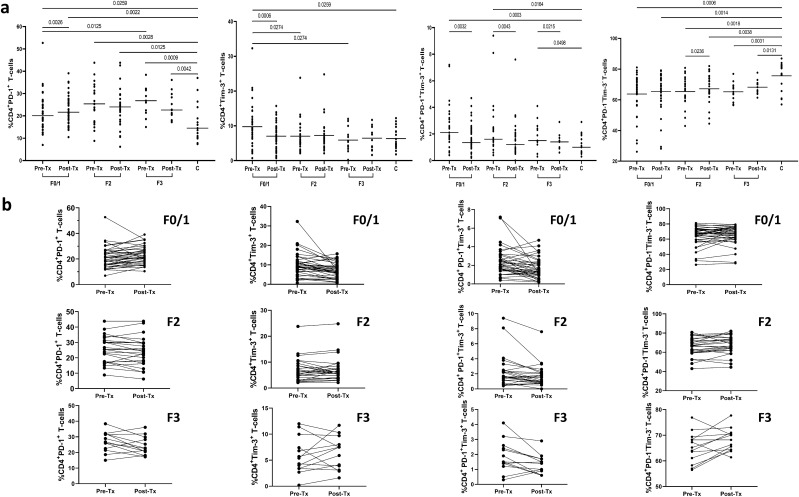
Peripheral blood CD4^+^ T-cells expression of PD-1 and Tim-3 in patients with different liver fibrosis scores (F0/1, F2, F3) before and six months after successful therapy for chronic HCV infection and in 18 non-infected controls (**a**) and individual PD-1 and Tim-3 expression changes before and after treatment (**b**). Horizontal lines represent median values. Numbers above each brace express P-values. *Pre-Tx* before therapy, *Post-Tx* after therapy, *C* non-infected controls.

**Figure 6:**
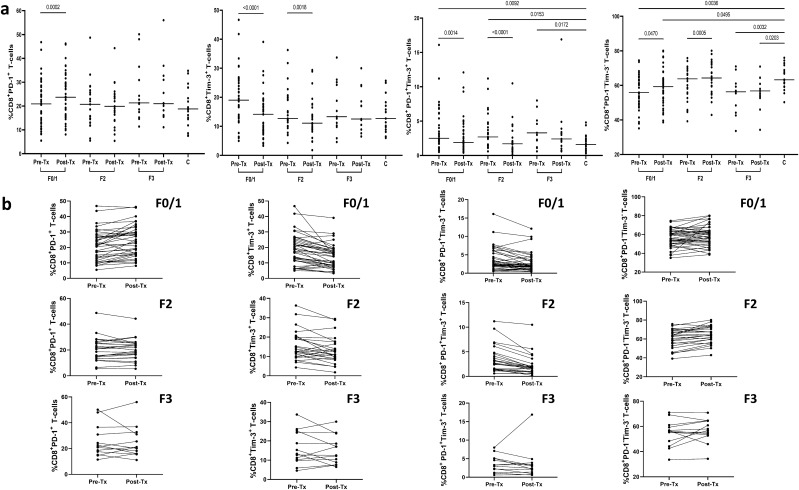
Peripheral blood CD8^+^ T-cells expression of PD-1 and Tim-3 in patients with different liver fibrosis stage (F0/1, F2, F3) before and six months after successful therapy for chronic HCV infection and in 18 non-infected controls (**a**) and individual PD-1 and Tim-3 expression changes before and after treatment (**b**). Horizontal lines represent median values. Numbers above each brace express P-values. *Pre-Tx* before therapy, *Post-Tx* after therapy, *C* non-infected controls.

Pretreatment exhaustion markers expression correlated with liver fibrosis score: the more advanced fibrosis, the higher percentage of CD4^+^PD-1^+^ T-cells (median 20.1% (range 7.0–52.7%) in F0/1 *vs* 25.4% (8.8–43.8%) in F2 *vs* 26.8% (15.0–38.4%) in F3, P = 0.0125). However, in every fibrosis stage the pretreatment percentage of CD4^+^PD-1^+^ T-cells was higher than in healthy controls (Fig. 5). In contrast, the more advanced fibrosis, the lower the frequency of CD4^+^Tim-3^+^ T-cells (9.0% (0.7–32.3%) in F0/1 *vs* 5.7% (2.1–23.8%) in F2 *vs* 5.4% (0.2–12.0%) in F3, P = 0.0274). When compared to controls, only F0/1 patients demonstrated higher pretreatment percentage of CD4^+^Tim-3^+^ T-cells (Fig. 5). While pretreatment percentages of CD4^+^PD-1^+^Tim-3^+^ T-cells, and CD8^+^PD-1^+^Tim-3^+^ T-cells in all three fibrosis groups were significantly higher than in healthy controls (Figs. 5 and 6), there were no differences between patients with different fibrosis stage. CD4^+^PD-1^−^Tim-3^−^ and CD8^+^PD-1^−^Tim-3^−^ T-cells percentages were similar in patients displaying different fibrosis scores but were significantly lower than in healthy controls with the exception of CD8^+^PD-1^−^Tim-3^−^ T-cells in F2 group which were similar to those in controls (Figs. 5 and 6). For the remaining T-cell subpopulations (CD8^+^PD-1^+^, CD8^+^Tim-3^+^) there were no statistically significant differences between different fibrosis stages, and the values were similar to those of healthy controls (Figs. 5 and 6).

Importantly, the more advanced fibrosis, the less likely it was that treatment would change the proportions of cells expressing exhaustion markers. Thus, F0/1 patients experienced increase in CD4^+^PD-1^+^ T-cells from 20.1% (7.0–52.7%) to 21.6% (10.5–39.1%), P = 0.0026 and CD8^+^PD-1^+^ T-cells from 20.9% (5.5–46.8%) to 23.7% (8.1–46.2%), P = 0.0002, and increase in CD8^+^PD-1^−^Tim-3^−^ T-cells from 55.8% (35.1–74.5%) to 59.3 (38.6–80.1%), P = 0.0470 and decrease in CD4^+^Tim-3^+^ T-cells from 9.0% (0.7–32.3%) to 6.8% (0.7–15.7%), P = 0.0006 and in CD8^+^Tim-3^+^ T-cells from 19.0% (5.0–46.7%) to 14.1% (3.3–39.1%), P < 0.0001, and decrease in CD4^+^PD-1^+^Tim-3^+^ T-cells from 2.1% (0.4–7.2%) to 1.3% (0.2–4.7%), P = 0.0032 and in CD8^+^PD-1^+^Tim-3^+^ T-cells from 2.5% (0.5–16.1%) to 1.9% (0.4–12.1%), P = 0.0014 (Figs. 5 and 6). In contrast, F3 patients displayed no significant changes in frequencies of cells expressing PD-1 and/or Tim-3 with the exception of CD4^+^PD-1^+^Tim-3^+^ T-cells, which decreased from 1.5% (0.3–4.1%) to 1.4% (0.6–2.9%), P = 0.0215. Patients with stage F2 fibrosis had less pronounced changes than F0/1 patients including decrease in PD-1^+^Tim-3^+^ T-cells (both CD4^+^ (1.6% (0.4–9.4%) *vs* 1.2% (0.0–7.6%), P = 0.0043) and CD8^+^ (2.7% (0.6–11.2%) *vs* 1.7% (0.2–10.5%), P =  < 0.0001) subpopulations, decrease in CD8^+^Tim-3^+^ T-cells (12.7% (4.3–36.3%) *vs* 11.1% (1.8–29.4%), P = 0.0018), decrease in CD4^+^PD-1^−^Tim-3^−^ T-cells (66.9% (43.0–80.8%) *vs* 66.7% (44.5–82.0%), P = 0.0236) and increase in CD8^+^PD-1^−^Tim-3^−^ T-cells (63.8% (39.2–75.8%) *vs* 64.3% (42.9–80.1%), P = 0.0005) (Figs. 5 and 6).

After treatment, none of the analyzed populations was significantly different between the subgroups of patients with different fibrosis stage.

Despite treatment-induced changes, some of the subpopulations did not reach values seen in healthy controls. These included higher CD4^+^PD-1^+^ T-cells in F0/1 (P = 0.0022), F2 (P = 0.0125) and F3 (P = 0.0042) groups as well as lower CD4^+^PD-1^−^Tim-3^−^ T-cells in F0/1 (P = 0.0014), F2 (P = 0.0038) and F3 (P = 0.0131) groups and lower CD8^+^PD-1^−^Tim-3^−^ T-cells in F0/1 (P = 0.0495) and F3 (P = 0.0203) groups (Figs. 5 and 6).

### Successful treatment affects HCV-specific CD8^+^ T-cell frequencies but not their PD-1 and Tim-3 expression phenotype

**Figure 7:**
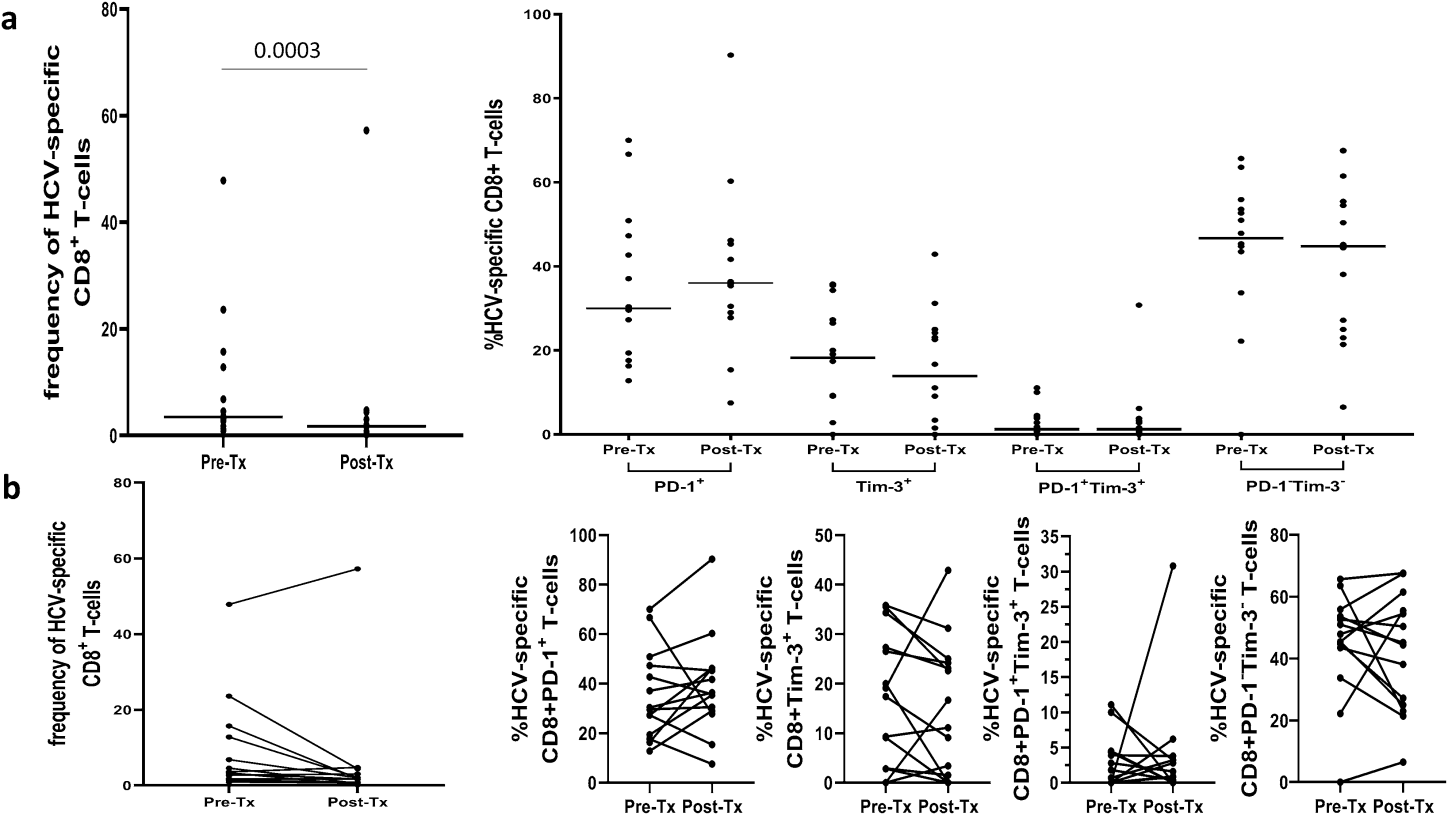
Peripheral blood HCV-specific CD8^+^ T-cells frequency and expression of PD-1 and Tim-3 in 32 patients before and six months after successful therapy of chronic HCV infection (**a**) and individual PD-1 and Tim-3 expression changes before and after treatment (**b**). Horizontal lines represent median values. Numbers above each brace express P-values. *Pre-Tx* before therapy, *Post-Tx* after therapy.

**Figure 8:**
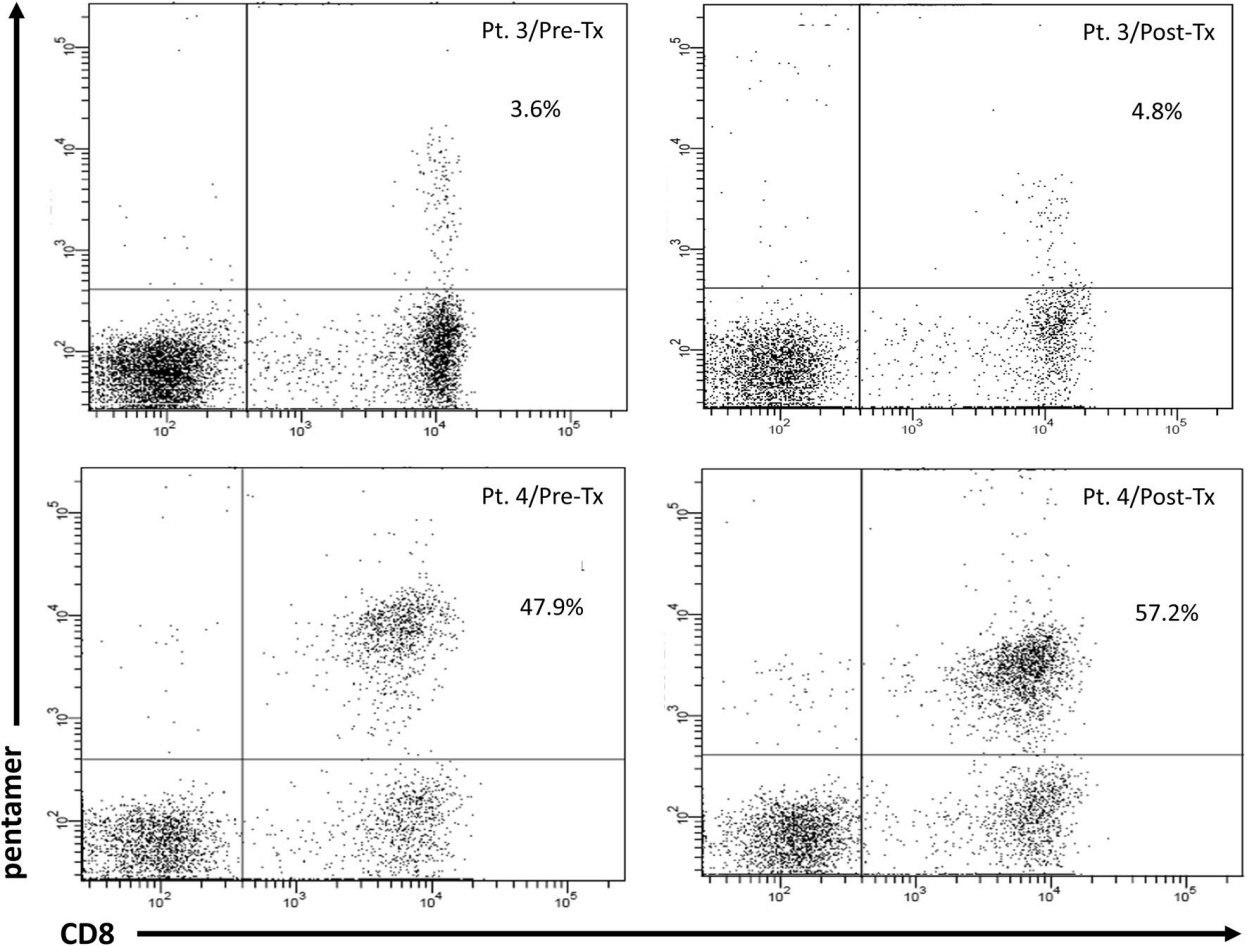
Representative cytometric analysis of peripheral blood HCV-specific CD8^+^ T-cells before and after successful therapy for chronic HCV infection in Patient 3 (Pt. 3) and Patient 4 (Pt. 4). *Pre-Tx* before therapy, *Post-Tx* after therapy.

Assessment of HCV-specific CD8^+^ T-cells frequencies and their exhaustion phenotype was feasible in 32 patients with HLA-A*02 allele. As shown in Fig. 7, treatment resulted in lowering the frequency of these cells from median 2.9% (range 0.1–47.9%) to 0.7% (0–57.2%), P = 0.0003. A representative cytometric analysis of treatment-related changes in HCV-specific CD8^+^ T-cells frequency in two patients is shown in Fig. 8.

While treatment resulted in some changes in the phenotype of HCV-specific cells, these differences did not reach statistical significance (Fig. 7). Before the therapeutic intervention, HCV-specific T-cells expressed PD-1 less frequently than after the treatment (30.0% (12.8–70.0%) *vs* 36.0% (7.5–90.3%)), were more likely to be Tim-3^+^ ((18.2% (0.0–35.8%) *vs* 13.9% (0.0–42.9%)), equally likely to be Tim-3^+^PD-1^+^ ((1.2% (0.0–11.1%) *vs* 1.2% (0.0–30.8%)) and more likely to be PD-1^−^Tim-3^−^ ((46.7% (0–65.7%) *vs* 44.8% (6.5–67.6%)).

### The effect of treatment scheme on the peripheral HCV-specific CD8^+^ PD-1^+^ T-cells frequency

We analyzed the effect of treatment with two different protocols (i.e., ledipasvir + sofosbuvir *vs* ombitasvir + paritaprevir + ritonavir + dasabuvir) correcting for variables differently distributed between the two treatment groups. Patients treated with ledipasvir + sofosbuvir displayed lower liver fibrosis score (59.3% *vs* 27.3% with F0/1, 40.7% *vs* 72.7% with F2/3, P = 0.0216) and higher age (median 61 (range 25–88) *vs* 49.5 (29–78), P = 0.0172). We found that changes in the percentages of cells expressing exhaustion markers were not different for these two protocols, the only exception being HCV-specific CD8^+^PD-1^+^ T-cells. Thus, while treatment with ledipasvir + sofosbuvir resulted in increase in percentage of HCV-specific CD8^+^PD-1^+^ T-cells from 28.4% (12.8–70.0%) to 40.8% (15.4–90.3%), treatment with ombitasvir + paritaprevir + ritonavir + dasabuvir resulted in their decrease from 39.9% (17.6–66.7%) to 31.7% (7.5–41.7%) (regression coefficient -39.41% 95% CI -63.71 to -15.11%, P = 0.005). The opposite was found for HCV-specific CD8^+^Tim-3^+^ T-cells: treatment with ledipasvir + sofosbuvir resulted in decrease in percentage of HCV-specific CD8^+^Tim-3^+^ T-cells from 18.7% (0–35.80%) to 6.2% (0–31.2%), while treatment with ombitasvir + paritaprevir + ritonavir + dasabuvir resulted in their increase from 14.2% (0–26.5%) to 20.4% (11.1–42.9%). However, these results were not significant in multivariate analysis (P = 0.066). Similarly, treatment with ledipasvir + sofosbuvir resulted in decrease in percentage of HCV-specific CD8^+^PD-1^−^Tim-3^−^ T-cells from 46.7% (0–65.7%) to 41.6% (6.5–67.6%), while treatment with ombitasvir + paritaprevir + ritonavir + dasabuvir resulted in their increase from 43.6% (22.2–55.9%) to 49.9% (21.4–67.5%). However, these results were also not significant in multivariate analysis (P = 0.063).

### Successful treatment diminishes IL-10 levels in plasma

**Figure 9:**
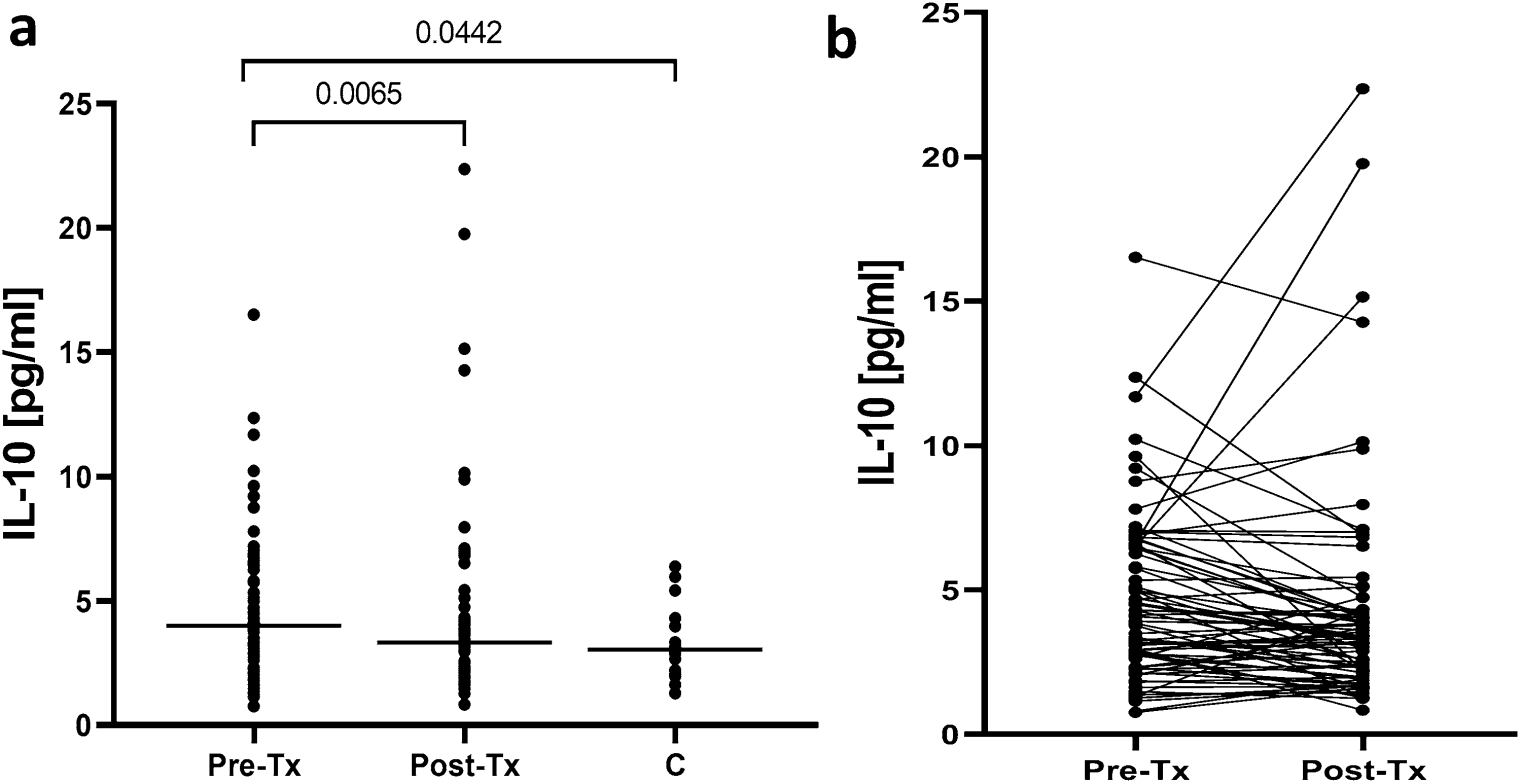
Plasma levels of IL-10 in 76 patients before and six months after therapy for chronic HCV infection and in 18 non-infected controls (**a**) and individual IL-10 changes before and after treatment (**b**). Horizontal lines represent median values. Numbers above each brace express P-values. *Pre-Tx* before therapy, *Post-Tx* after therapy, *C* non-infected controls.

Before therapy, patients displayed significantly higher plasma IL-10 levels than controls (median 4.0 (range 0.8–16.5) pg/mL *vs* 3.0 (1.3–6.4) pg/mL, P = 0.0442) (Fig. 9). Treatment resulted in decrease of IL-10 levels to 3.3 (0.8–22.4) pg/mL, P = 0.0065, which were now similar to the levels observed in controls.

**Figure 10:**
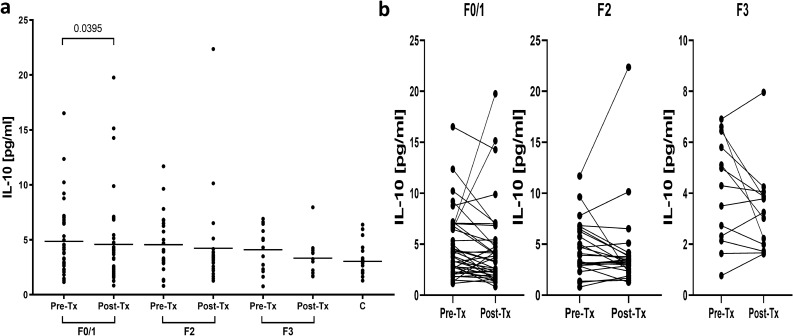
Plasma levels of IL-10 in 76 patients in groups of patients displaying different liver fibrosis scores (F0/1, F2, F3) before and six months after therapy for chronic HCV infection and in 18 non-infected controls (**a**) and individual IL-10 changes before and after treatment (**b**). Horizontal lines represent median values. Numbers above each brace express P-values. *Pre-Tx* before therapy, *Post-Tx* after therapy.

Plasma IL-10 levels were not significantly different between patients displaying different fibrosis scores neither before (4.0 (1.1–16.5) pg/mL in F0/1 *vs* 3.9 (0.8–11.7) pg/mL in F2 *vs* 4.3 (0.8–6.9) pg/mL in F3), nor after treatment (3.5 (0.8–19.8) pg/mL in F0/1 *vs* 3.3 (1.3–22.4) pg/mL in F2 *vs* 3.2 (1.6–8.0) pg/mL in F3). Plasma IL-10 levels were also not significantly different between patients with different fibrosis stage and healthy controls, neither before nor after treatment. However, a significant decrease of IL-10 levels after treatment was observed but was limited to F0/1 patients (P = 0.0395) (Fig. 10).

## Discussion

So far relatively few studies reported on the effects of treatment-induced HCV elimination on the immune exhaustion status of T-cells, and especially on HCV-specific cells. Furthermore, these studies were often inconclusive, carried out on small cohorts of patients infected with various HCV genotypes, assessed different immunological parameters, confined to the analysis of only one inhibitory T-cell receptor, or were conducted on patients treated with IFN or even experimental drugs, which were eventually abandoned^47–54^.

The current standard of care of chronic HCV infection advocates the use of DAA, which are successful in over 95% of patients^55^. However, their effect on the immune exhaustion status of T-cells has not been analyzed in detail, especially with respect to CD4^+^ T-cell population. Importantly, it is the restoration of antiviral immunity, manifested by the reversal of the exhausted T-cells phenotype, that may be critical for successful treatment, since the presence of HCV-RNA in serum at the end of DAA-based therapy does not preclude sustained virologic response (SVR)^56,57^.

Similar to other authors, we found that chronic HCV infection is associated with increased expression of exhaustion markers on peripheral blood CD4^+^ and CD8^+^ T-cells as compared to non-infected controls^28,58^. Furthermore, in our study CD4^+^ and CD8^+^ T-cells expressing Tim-3 and co-expressing PD-1 and Tim-3 decreased after successful treatment and this was accompanied by increased frequency of PD-1^−^Tim-3^−^ CD4^+^ and CD8^+^ T-cells, suggesting that the immune exhaustion induced by prolonged infection may be at least partially reversed once the infecting pathogen is eliminated. A number of mechanisms could facilitate this reversal of functional T-cells exhaustion such as rapid reduction of viral burden, up-regulation of soluble factors involved in T-cell activation, elimination of viral proteins known to inhibit innate immune responses and reduction in the production of immunosuppressive cytokines including IL-10^59–61^. Indeed, in our study IL-10 plasma values returned to near normal levels after the elimination of HCV. While increase of IL-10 blood levels in chronic HCV infection is likely to be driven by chronic inflammation and immune regulatory mechanisms^40^, HCV proteins could also stimulate IL-10 production directly^62^.

In contrast to Tim-3 expression and PD-1 + Tim-3 co-expression, the frequencies of CD4^+^ T-cells expressing PD-1 did not change significantly after treatment and the CD8^+^PD-1^+^ population even increased. Similar observation was reported by Zhang et al.^54^ who found no significant differences in the population of CD4^+^ T-cells expressing PD-1 before and after DAA-induced SVR. The mechanisms behind this phenomenon are unclear. Since PD-1 expression may vary within particular subsets of CD4^+^ and CD8^+^ T-cells and is increased during early and intermediate cell differentiation stages^63^, high post-treatment PD-1 expression among our patients could have been the result of a switch to some earlier differentiation stage of T-cell populations once the infection has been cleared. Alternatively, the relative stability or increase of PD-1^+^ T-cell subpopulations may have been the result of PD-1^+^Tim-3^+^ cells becoming single positive PD-1^+^ cells.

Earlier studies conducted on HIV-infected patients suggested that PD-1 is a marker of early T-cell exhaustion representing a stage of impaired proliferation but still relatively well preserved function manifested in the ability of cytokine synthesis^64^, whereas Tim-3 is a marker of more advanced T-cell exhaustion associated with cytokine synthesis impairment and susceptibility to apoptosis^27,65^. Furthermore, it was previously proposed that co-expression of PD-1 and Tim-3 receptors characterizes the most exhausted and dysfunctional T-cell subset^27^. This view is compatible with the observation that the frequency of PD-1^−^Tim-3^−^ HCV-specific T-cells is much higher than those of PD-1^+^Tim-3^+^ phenotype in patients with acute resolving infection but not in patients in whom acute infection progressed to chronicity^17^. Similarly, in a study employing a murine LCMV infection model, dual expression of these iRs was associated with progression to chronic infection^27^. Our finding of uncommon co-expression of these two markers is also congruent with the existence of a hierarchical model of exhaustion, in which this process is initiated by PD-1 expression, followed by Tim-3 and finally by co-expression of the latter two markers.

In our study we made a novel observation that the pretreatment PD-1 and Tim-3 phenotypes of T-cells correlated with liver fibrosis scores. Thus, the more advanced fibrosis, the higher the frequency of CD4^+^PD-1^+^ and the lower the frequency of CD4^+^Tim-3^+^ cells. Given the hierarchical model of T-cell exhaustion mentioned earlier, it is likely that in advanced long-lasting infection reflected by pronounced fibrosis, a substantial proportion of Tim-3^+^ cells may have already been deleted. Furthermore, we found that in patients with advanced fibrosis, successful treatment was not followed by changes in the populations of cells expressing exhaustion markers. Thus, while F0/1 patients experienced an increase in the frequencies of PD-1^+^ CD4^+^ and CD8^+^ and PD-1^−^Tim-3^−^ CD8^+^ T-cells and decrease in the frequencies of Tim-3^+^ CD4^+^ and CD8^+^ T-cells and PD-1^+^Tim-3^+^ CD8^+^ T-cells, this was not the case in F3 patients. Similarly, successful DAA treatment was associated with a significant lowering of IL-10 levels in plasma only in F0/1 patients, having no effect in F2 or F3 patients. Therefore, it seems that advanced liver fibrosis marks patients with advanced and poorly reversible immune-related changes. Vranjkovic et al.^66^ found that HCV-infected patients with pronounced liver fibrosis (F4) displayed hyperfunctional activity of peripheral CD8^+^ T-cell subsets sustained up to a year after treatment, and the impact of successful DAA therapy on T-cell activation depended largely on the stage of liver fibrosis. Furthermore, in F4 individuals DAA therapy had no effect on elevated concentrations of systemic inflammatory cytokines and decreased levels of inhibitory TGF-β in plasma. These data suggest that HCV-infected patients with advanced liver disease have a long-lasting and irreversible immune exhaustion. However, it is unclear whether this effect is due to fibrosis itself or rather to a long-lasting infection of which fibrosis is only a manifestation, as the length of infection in the majority of patients could not be determined.

In our patients HCV-specific CD8^+^ T-cells frequency was significantly lower after treatment, which was likely due to the elimination of these cells after HCV clearance. Similarly, Han et al.^49^ observed that HCV-specific CD8^+^ T-cells, including antigen-experienced (KLRG1^+^CCR7^−^) HCV-specific CD8^+^ T-cell subset, decreased after SVR. We also found that the expression of PD-1 on HCV-specific CD8^+^ T-cells was more frequent while that of Tim-3 less frequent after treatment, but these differences did not reach statistical significance. Aregay et al.^67^ demonstrated that HCV-specific CD8^+^ T-cell function was not restored following HCV eradication by means of DAA treatment, since expression of CD5, LAG-3, PD-1 and Tim-3 on HCV-specific CD8^+^ T-cells, impaired IFN-ϒ and MIP-1β production, metabolic deregulation and mitochondrial dysfunction did not change. These findings imply that in chronically infected patients, HCV-specific CD8^+^ T-cells exhaustion phenotype may not be restored following DAA-induced viral eradication and suggest that a reversal of this phenotype, similar to that observed in spontaneous viral clearance, is not achievable. Thus, it is likely that chronic stimulation with HCV antigens is related to irreversible changes in the HCV-specific CD8^+^ T-cell population.

In our study the type of DAA treatment received was found to affect exhaustion markers of HCV-specific CD8^+^ T-cells differently. In particular, treatment with ledipasvir + sofosbuvir led to an increased expression of PD-1 whereas treatment with ombitasvir + paritaprevir + ritonavir + dasabuvir resulted in decreased expression of PD-1^+^. The reason for this phenomenon is unclear and the only difference between the two treatments was different number of drug targets. While ledipasvir + sofosbuvir are NS5A and NS5B inhibitors, respectively, ombitasvir + paritaprevir + ritonavir + dasabuvir are NS5A, NS3/4A/CYP3A4 and NS5B inhibitors, respectively. Shrivastava et al.^68^ reported that the most complete restoration of HCV-specific immune response in HIV/HCV coinfected patients was observed in those treated with a regimen that inhibits three distinct stages of the HCV life cycle.

Interestingly, in our study older age was associated with the presence of higher percentage of CD4^+^PD-1^+^ and CD8^+^PD-1^+^Tim-3^+^ T-cells. This can be due to the direct effect of age or it could reflect the duration of infection^69^. Similarly, male sex was related to higher IL-10 plasma levels and higher percentage of CD8^+^PD-1^+^ T-cells. The reason probably lays in the fact that immunity in males is characterized by weaker humoral and cellular immune responses and this could be partly due to estrogen and testosterone effects on immunity^70^.

## Conclusions

In summary, we found that a successful therapy of chronic HCV infection with DAA lowered expression of T-cell exhaustion markers to near normal values and reduced IL-10 levels in plasma, but these changes were largely confined to patients with minimal or no liver fibrosis. DAA treatment was found to lower HCV-specific CD8^+^ T-cells frequency but had little effect on the expression of exhaustion markers by these cells which suggests that long-term antigenic stimulation results in irreversible changes to the HCV-specific T-cell compartment.

## Methods

### Patients and controls

Seventy-six patients with chronic HCV infection (47 women and 29 men, median age 58.5 years, range 25–88), who underwent anti-HCV therapy at the Warsaw Hospital for Infectious Diseases Outpatient Clinic in the years 2016–17 were studied prospectively. All were HCV- RNA positive for at least 6 months prior to therapy, and all were infected with HCV genotype 1 (genotype 1b was present in 74 and genotype 1a was present in two patients). Fifty-four patients were treated with ledipasvir and sofosbuvir (Harvoni, Gilead Sciences Inc, Foster City, CA, USA) 90 and 400 mg per day, respectively, and 22 patients received ombitasvir, paritaprevir, ritonavir (Viekirax, AbbVie Inc., Lake Bluff, IL, USA) at doses of 25, 150, and 100 mg per day, respectively, along with dasabuvir (Exviera, AbbVie Inc.) 500 mg per day.

Treatment was administered for 12 weeks in 48 patients, whereas in 19 patients receiving Harvoni and in 9 patients receiving Viekirax and Exviera, the duration of therapy was 8 weeks. Clinical effectiveness of treatment was assessed 6 months post-treatment using a quantitative PCR test (Abbott RealTime HCV Viral Load Assay, Abbott Laboratories, Abbott Park, IL, USA; sensitivity 12 IU/mL). Sustained virologic response (SVR) was achieved in all 76 patients (100%).

Eighteen healthy anti-HCV negative volunteers served as control group. Characteristics of the study and control groups are presented in Table 1.

Thirty-six mL of EDTA-anti-coagulated whole blood was collected from all patients before and 6 months post-treatment while blood from control subjects was collected only once.

The study protocol followed ethical guidelines of the 2013 Declaration of Helsinki and was approved by the Bioethical Committee of the Medical University of Warsaw (Approval Number KB/77/A/2015). All patients and controls provided written informed consent.

### PBMC staining and flow cytometric analysis

Peripheral blood mononuclear cells (PBMC) and plasma were separated from whole blood using Lymphoprep reagent (Stemcell Technologies Inc, Vancouver, British Columbia, Canada). Plasma samples were stored at – 80 °C while PBMC were analyzed immediately after isolation.

### HLA-A*02 typing

The presence of HLA-A*02 allele was verified by flow cytometry using Mouse Anti-Human HLA-A2 Clone BB7.2 antibody (BD Pharmingen, San Diego, CA, USA) and by quantitative PCR as described elsewhere^71^.

### T-cell phenotyping

Isolated PBMC were resuspended in PBS pH 7.2 (Life Technologies, Carlsbad, USA), stained with BD Horizon Fixable Viability Stain 780 (BD Biosciences, San Diego, CA, USA) and subsequently mixed with FcR blocking reagent (Miltenyi Biotec, Bergisch Gladbach, Germany) following manufacturer's protocol. Next, one million cells were resuspended in Stain Buffer with 0.2% (w/v) bovine serum albumin (BD Pharmingen). mixed with 5 µl of BV421 Mouse Anti-Human Tim-3 (CD366) Clone 7D3 (BD Horizon, San Diego, CA, USA), 5 µl of Alexa Fluor 647 Mouse Anti-Human PD-1 (CD279) Clone EH12.1, 5 µl of PerCP-Cy 5.5 Mouse Anti-Human CD3 Clone UCHT1, (both from BD Pharmingen), 5 µl of V500 Mouse Anti-Human CD4 Clone RPA-TY (BD Horizon) and 1 µl of Mouse Anti-Human CD8 FITC Clone LT8 (ProImmune Oxford, United Kingdom). Cells with added antibodies were incubated for 20 min at 4 °C. After incubation, stained cells were washed twice with PBS pH 7.2 (Life Technologies) and resuspended in 300 μL of Stain Buffer.

Controls included unstained cells and isotype controls consisting of cells stained with 5 µl of Mouse Anti-Human IGG1 Alexa Fluor 647 and 2.5 µl of Mouse Anti-Human IGG1 BV421 instead of Alexa Fluor 647 Mouse Anti-Human PD-1 (CD279) and BV421 Mouse Anti-Human Tim-3 (CD366), respectively (both from BD Pharmingen). For data acquisition, one million stained cells were used. The results were acquired immediately after staining by BD FACS Canto II Flow Cytometer (BD Biosciences), using BD FACS Diva version 6.0 program (BD Biosciences).

### HCV-specific CD8^+^ T-cells enrichment and phenotyping

HCV-specific CD8^+^ T-cell populations are difficult to detect directly^72^. We employed a combination of MHC multimer staining, magnetic-bead enrichment, and multiparametric flow cytometry for ex vivo detection and characterization of rare antigen-specific CD8^+^ T-cells^73,74^. In 32 HLA-A*02^+^ patients (genotype 1b HCV infection), 25 million of PBMC were subjected to this procedure. In brief, custom PE-labeled Pro5 Recombinant MHC class I Pentamer containing HLA-A*02-restricted HCV NS3_1406_ immunodominant epitope KLSGLGLNAV (corresponding to genotype 1b) (ProImmune) was added to cells resuspended in Stain Buffer and incubated for 10 min at room temperature in the dark. The cell suspension was then washed with MACS Buffer (Miltenyi Biotec) and cell pellet was resuspended with Anti-PE Micro Beads (Miltenyi Biotec) and MACS Buffer and incubated for 20 min at 4 °C, protected from light. Next, cells were washed twice with MACS Buffer and passed through a 70 μm Cell Strainer (BD Biosciences). Magnetic MS Columns (Miltenyi Biotec) were used for cell separation following manufacturer’s instructions. Enriched cells were counted and stained with anti-CD3, -CD4, -CD8, -PD-1, -Tim-3 antibodies for 20 min at 4 °C as described above.

### Efficiency of the HCV-specific T-cell enrichment

Without the enrichment, CD8^+^ specific T-cells were rarely detectable at measurable numbers in our patients (detectable in 14 (43.7%) of cases at mean frequency of 0.05% of total CD8^+^ T-cells before treatment and in 18 (56.2%) of cases at mean frequency of 0.07% of total CD8^+^ T-cells after treatment). However, using the enrichment procedure, detectability increased to 32 of cases (100%) and the average frequency increased 136-fold (to 6.8% of total CD8^+^ T-cells) before treatment and to 29 of cases (90.6%) and the average frequency increased 41.4-fold (to 2.9% of total CD8^+^ T-cells) after treatment.

### Cytometric data analysis

For data analysis, the initial gate was set on lymphocytes on the forward scatter (FSC) *vs* side scatter (SSC) dot plot. Subsequently, singlet cells gate was set on FSC-H versus FSC-A dot plot. Next, based on SSC *vs* APC-Cy7 dot plot, only live cells were gated. Additionally, the following gates were employed: CD3^+^, CD4^+^, CD8^+^, pentamer^+^, PD-1^+^, Tim-3^+^, PD-1^+^Tim-3^+^ and PD-1^−^Tim-3^−^.

### IL-10 plasma levels measurement

IL-10 levels were measured in plasma by ELISA (Human IL-10 ELISA Max Kit; BioLegend, San Diego, CA, USA) following manufacturer's protocol. The ELISA Analysis program available from www.elisaanalysis.com was used to calculate IL-10 concentrations in plasma, expressed as pg/mL.

### Statistical analysis

Percentages of gated cell populations and IL-10 levels were expressed as median (range). Wilcoxon matched-pairs signed ranks test was used to compare percentages of T-cells expressing exhaustion markers and IL-10 levels before and after treatment while Mann–Whitney U test was used to compare patients with controls. Kruskall-Wallis test was used to compare T-cell exhaustion markers and IL-10 levels between groups with different stage of fibrosis. A general linear model (GLM) was used to test independent pretreatment factors and treatment scheme on percentages of T-cells expressing exhaustion markers and IL-10 levels in plasma. All P values were two-tailed and considered significant when ≤ 0.05.
